# Reproducibility of NMR Analysis of Urine Samples: Impact of Sample Preparation, Storage Conditions, and Animal Health Status

**DOI:** 10.1155/2013/878374

**Published:** 2013-06-23

**Authors:** Christina Schreier, Werner Kremer, Fritz Huber, Sindy Neumann, Philipp Pagel, Kai Lienemann, Sabine Pestel

**Affiliations:** ^1^numares GROUP, Josef Engert Straße 9, 93053 Regensburg, Germany; ^2^Institute of Biophysics and Physical Biochemistry and Centre of Magnetic Resonance in Chemistry and Biomedicine, University of Regensburg, 93040 Regensburg, Germany; ^3^Department of Computer Science, Technical University Dortmund, Otto-Hahn-Straße 16, 44221 Dortmund, Germany; ^4^Group General Pharmacology, Department Drug Discovery Support, Boehringer-Ingelheim Pharma GmbH & Co. KG, 88397 Biberach an der Riss, Germany

## Abstract

*Introduction.* Spectroscopic analysis of urine samples from laboratory animals can be used to predict the efficacy and side effects of drugs. This employs methods combining ^1^H NMR spectroscopy with quantification of biomarkers or with multivariate data analysis. The most critical steps in data evaluation are analytical reproducibility of NMR data (collection, storage, and processing) and the health status of the animals, which may influence urine pH and osmolarity. *Methods.* We treated rats with a solvent, a diuretic, or a nephrotoxicant and collected urine samples. Samples were titrated to pH 3 to 9, or salt concentrations increased up to 20-fold. The effects of storage conditions and freeze-thaw cycles were monitored. Selected metabolites and multivariate data analysis were evaluated after ^1^H NMR spectroscopy. *Results.* We showed that variation of pH from 3 to 9 and increases in osmolarity up to 6-fold had no effect on the quantification of the metabolites or on multivariate data analysis. Storage led to changes after 14 days at 4°C or after 12 months at −20°C, independent of sample composition. Multiple freeze-thaw cycles did not affect data analysis. *Conclusion.* Reproducibility of NMR measurements is not dependent on sample composition under physiological or pathological conditions.

## 1. Introduction

The quality and reproducibility of any given analytical process is strongly dependent on the quality of the samples studied. This can, in turn, be influenced by several factors, especially sample collection, storage, and handling procedures. Although these effects are well-known, only relatively few studies have been performed to date to quantify their effect [1–6]. 

In general, chemical degradation processes, such as oxidation and decomposition of chemically unstable sample components, can severely impact sample composition. Biological degradation due to microbial contamination may occur additionally in biological and organic samples, especially when collected under non-aseptic conditions (e.g., urine collection using metabolic cages). Further, biological samples may have different compositions not only due to the “normal” interindividual variations reflecting the individuals in a cohort, but also due to external factors, such as the metabolic, nutritional, or health status of an animal. To an even greater degree, pharmacological effects may impact sample composition. For example, treatment of animals with diuretic or antidiuretic drugs or nephrotoxicants modifies the salt concentration, pH value, and protein content of urine samples [7–10]. All these factors may contribute to the speed and the quality of sample transformation occurring *ex vivo*. If changes in these parameters are not taken into account during sample preparation, they may affect the nuclear magnetic resonance (NMR) analysis of a single metabolite. Only relatively few recommendations for sample collection, preparation, and storage have been published, especially on stabilization of the pH, which is the most important parameter in ^1^H NMR spectroscopy [1–3]. Other studies give recommendations for buffer compositions to stabilize plasma [4], bile, and urine samples [1] or urine samples only [11], or for the maximum time for storing samples at 4°C [2, 5] or room temperature (RT) [6]. Apart from these established standard procedures, little is known about the degradation processes which occur during sample storage and processing. While biological and chemical degradation processes show little effect on inorganic ions, such as sodium or potassium, the concentrations of organic metabolites are affected more often. Additionally changes in sample composition, for example, after treatment with pharmaceuticals, should be considered.

The extent of changes caused by the specific sample composition due to pH, salt content, or sample decomposition, in the following referred to as biotransformation, may also lead to chemical shift differences in ^1^H NMR spectroscopy; the magnitude of effect is largely unknown. Hence, sample biotransformation may severely affect evaluation, especially when included in (unsupervised) metabonomic investigations, since data analysis methods for metabonomic studies need to be based on large and reliable databases. This means that one of the most important factors for the reliability of the results is the quality and the stability of the sample and the subsequent exclusion of method-related artefacts. High analytical reproducibility can only by achieved, when the influence of sample variation on chemical shift can be calculated and excluded. 

The aim of this study is to characterize the possible effects of urine sample modification in rats, and to use this information to improve the quality of data analysis for single metabolite quantification and for future metabonomic studies. Rats were treated in this study with natrosol (Natrosol^®^, hydroxyethylcellulose), with the loop diuretic furosemide (Lasix^®^) or with the nephrotoxicant hexachlorobutadiene (HCBD). As natrosol is one of the standard placebos used in (safety) investigations of pharmaceutical compound candidates, the urine samples of rats treated with natrosol should represent a physiological urine composition [13]. The diuretic furosemide inhibits the Na-K-2Cl symporter in the thick ascending limb loop of Henle in the kidney, and thereby increases salt and water excretion [7, 8, 10], resulting in diluted and salt-rich urine [9]. HCBD is a nephrotoxic tool compound [14, 15], which leads to increased urine protein concentrations and increased enzyme excretion (own unpublished data). Data acquisition and analysis in ^1^H NMR spectroscopy may not only be affected by variations of pH or degradation processes, but also by the salt content of samples which impacts the electron density and therefore the magnetic shielding of each proton. Thus, to achieve a sufficiently wide database, pH and salt concentrations of the urine samples of the three treatment groups were adjusted before mixing the samples with phosphate buffer. Moreover, the effects of sample storage, pH and salt concentrations were then analyzed for their potential impact on ^1^H NMR-based methods for metabolite quantification and on a metabonomic classification method. 

## 2. Materials and Methods

### 2.1. Materials

Natrosol^®^ 250 HX Pharm (Hydroxyethylcellulose Ph. Eur.) was supplied by Hercules (Düsseldorf, Germany), furosemide (F-4381, Lasix^®^) by Hoechst (Frankfurt/Main, Germany), hexachlorobutadiene (HCBD) by Sigma-Aldrich (Steinheim, Germany). DSS was purchased from Cambridge Isotope Laboratories (CIL) (Andover, MA, USA); D_2_O and 5 mm NMR tubes type Norell 502 from Euriso-Top (Gif-sur-Yvette, France).

### 2.2. Animals

Hannover Wistar rats CRL: WI (GIx/BRL/HAN)IGS BR of both sexes (as specified with the data) were obtained from Charles River (Sulzfeld, Germany). The rats weighed 180 to 210 g and were 8 to 9 weeks old. For at least 4 days prior to the experiments, the animals were housed on a 12-hour day/night rhythm (light from 6 a.m. to 6 p.m.), food (autoclaved standard rat chow from Provimi Kliba, Kaiseraugst, Switzerland) and water were provided ad libitum. Treatment of the animals followed the German Law on the Protection of Animals and was performed with permission of the state animal welfare committee.

### 2.3. Samples for Calibration and Accuracy Measurements

Analyte concentrations for calibration samples and for accuracy test samples were selected with the aim to cover physiological and pathological concentrations ranges in urine samples. These ranges were estimated from own unpublished data as measured with clinical chemistry methods. Normal ranges (mean ± standard deviation (SD)) and pathological ranges (minimum and maximum) were measured for glucose (normal range 124 ± 43 *μ*g/mL, pathological range 31 to 22317 *μ*g/mL), creatinine (normal range 194 ± 86 *μ*g/mL, pathological range 41 to 1161 *μ*g/mL) and urea (normal range 20871 ± 8271 *μ*g/mL, pathological range 407 to 98840 *μ*g/mL) (see Supplement Figure 1, available online at http://dx.doi.org/10.1155/2013/878374). Concentration data were not available in house for the other metabolites and were thus estimated at risk to be within the same coverage. This was supported by data from Saude et al. from human and guinea pig urine, giving mean values for citrate, creatinine and lactate between 0.22 to 9.03 mM [16] and by Slupsky et al. from human urine, giving mean values for citrate and creatinine of 350 to 2749 and 5107 to 14087 *μ*M, respectively [17].

*Calibration samples* for glucose, phenylacetylglycine (PAG), creatinine, lactate, citrate and urea were dissolved in purified water containing 0.01% sodium azide (NaN_3_). 20% (v/v) of a solution of DSS (20 mM) in D_2_O was added as an internal chemical shift reference. The system was calibrated using 10 *μ*g/mL, 100 *μ*g/mL, 1,000 *μ*g/mL, and 10,000 *μ*g/mL of each metabolite. 

A set of 100 randomly generated *accuracy test samples* (hereinafter given as *synthetic samples* in this publication) was generated for blind testing of the system for accuracy. The samples (prepared with mixed concentrations according to a computer-generated matrix) contained glucose, creatinine, lactate, and citrate within a continuous concentration range of 10 *μ*g/mL to 10,000 *μ*g/mL. 0.01% sodium azide (NaN_3_) and 20% (v/v) of a solution of DSS (20 mM) in D_2_O were added. The variations of sample composition resulted in different pH values (range pH 3–8), covering a range comparable to physiological/pathological urine samples (normal range 6.8 ± 1.1, pathological range minimum 4.0 and maximum 9.1, see Supplement Figure 2). 

### 2.4. Samples for Biological References, Testing of pH, Salt and Storage Effects

Rats received either natrosol (placebo), furosemide (diuretic) or HCBD (nephrotoxicant). Dose selection was based on information from the literature (natrosol: [13]; furosemide: [7, 8, 18]; HCBD: [14, 15]). Compounds were administered orally with a volume of 10 mL/kg. Urine was collected at 8 h and 24 h after administration of the compounds using metabolic cages, and the urine was cooled immediately to 4°C already during urine collection. The animals had free access to water but not to food during the entire experiment. Effects described in the literature were confirmed by our own clinical chemistry measurements: major effects for furosemide were an increase in urine volume and salt excretion (Na^+^, Cl^−^, K^+^) [9], and for HCBD an increase in urine enzyme and protein excretion (own unpublished data). Additionally, (lack of) nephrotoxicity was tested by metabonomics methods [19]. For the different evaluations, variable groups of rats were used as follows (Table 1).

*For the sample stability test*, urine from natrosol-, furosemide-, or HCBD-treated rats was pooled treatment-wise (5 males and 5 females per treatment group). The pools were then subdivided in aliquots for testing of storage effects alone or in combination with pH or salt effects. Therefore, adjustment of pH and increase of salt concentration was done directly after pooling the urine samples from natrosol-, furosemide-, or HCBD-treated rats. Despite this, we had only single point measurements for each effect, that is, each different kind of rat treatment and sample modification.

- *pH was adjusted* using 1 N HCl or 1 N NaOH, aiming at a range from pH 3 to pH 9. Values of pH were measured before and after addition of phosphate-buffer (Supplement Table 1) and covered a range of pH 2.90 to pH 9.15 before and pH 6.62 to pH 7.64 after addition of buffer.
- *Salt concentrations* were increased by adding NaCl solution (35 mmol/L) to reach about 20-fold increases of salt concentrations (based on typical NaCl concentrations in urine samples from natrosol-treated rats). Na^+^ and Cl^−^ values were determined in the original samples using the free ISE unit of the Konelab 60i (Thermo Fisher Scientific, Vantaa, Finland), but not in those after addition of NaCl because this was technically impossible. Therefore, osmolality was measured using the OSMOMAT^®^ auto (Gonotec GmbH, Berlin, Germany) as a surrogate parameter. For information, theoretical sample salt concentrations were calculated in the samples with added NaCl (Supplement Table 2).
- *Storage effects* were investigated with the samples prepared for determination of pH and salt effects. These samples were thawed under controlled conditions (see below) and measured for the first time. These so-called “day 0 data” were used as reference data for the refrigerating and the freezing experiments.
  1. To investigate *refrigerating effects* (storage at 4 to 8°C in a standard refrigerator from Bosch, Gerlingen-Schillerhöhe, Germany, type KGV33600, hereinafter given as 4°C), the samples were kept refrigerated after the first measurement, except for 30 min during each NMR analysis (25°C). 1D ^1^H NMR spectra were recorded on days 1, 2, 3, 4, 6, 8, 10, 12, 14, 17, 20, 23, 26, 29, 33, 37, 41, 45, and 49. These investigations were done with the “original” samples from natrosol-, furosemide-, and HCBD-treated rats, and all samples with pH- and salt-modifications (max. *n* = 18 per treatment group, with exclusion of samples with suggested microbial contamination). The data from single metabolite measurements were normalized to the “day 0 sample” of each treatment and sample modification, in order to depict the variability from storage over time on the abscissa, while variability from sample modification and multiple measures is depicted as standard deviation (SD).
  2. To investigate *freezing effects *(storage at −20 to −25°C in a standard freezer from AEG, Nürnberg, Germany, type A 80270-GT, hereinafter given as −20°C), new aliquots of the samples were thawed after storage periods of 1, 3, 6, 12, and 24 months to avoid effects caused by additional freeze-thaw cycles. These investigations were done with the original samples from natrosol-, furosemide-, and HCBD-treated rats, and with those modified pH to 5, 6, and 7.5 and increased salt concentrations with 3-, 6-, and 10-fold increases (*n* = 7 per treatment group). As for the refrigerating effects, the data from single metabolite measurements were normalized to the “day 0 sample”.
  3. To investigate *freeze-thaw effects*, separate samples were generated from *n* = 2 male animals as described above. Samples were submitted to 6 freeze-thaw cycles (−20°C/room temperature, with variation from 22 to 26°C), each thawing step followed by NMR analysis.

*Samples for depicting physiological variance/pharmacological effects* were taken from routine procedures after treatment of the rats with natrosol, furosemide or HCBD (group size *n* = 8, four male and four female rats). Physiological day-to-day variance was investigated in the natrosol-treated groups (4 groups from different treatment days), and pharmacological effects were covered in the groups treated with furosemide and HCBD. 

Original and manipulated urine samples were mixed with phosphate buffer (0.8 M Na_2_HPO_4_/NaH_2_PO_4_ pH 7.4 with 9% D_2_O and 50 *μ*M DSS), at a ratio of one part buffer with two parts urine. Thereafter, samples were divided into aliquots of 625 *μ*L in 2 mL polypropylene tubes, and immediately stored at −20°C. Samples were transported under dry ice conditions from the animal laboratory to the NMR laboratory. 

Sample thawing was done under a standard sample handling procedure to avoid effects of different thawing processes: the frozen polypropylene tubes were immediately immersed in a water bath (*v* = 2.0 L, maximum number of samples = 20) with a temperature of 18 ± 2°C for 10 min and then mixed, transferred to the NMR tube and stored at 4°C until ^1^H NMR measurement. 

### 2.5. ^1^H NMR Spectroscopy

^1^H NMR spectra were collected at 298 K on a Bruker AVANCE II^+^ spectrometer with 600.3 MHz operating proton frequency. The spectrometer was equipped with a second-generation digital receiver unit (2G-DR, digitizer mode was set to *baseopt*), a 5 mm inverse triple resonance *z*-axis gradient (TXI) probe and a Bruker automatic sample changer (BACS). For every urine sample a gradient 1D ^1^H NOESY experiment using presaturation during the relaxation delay (d1 = 2 s) and a mixing time of 8 ms was used. All spectra were recorded with 32 scans and a total recycling time of 3.36 s. The correct sample temperature during the sample changing process was automatically adjusted to within ±0.2 K of the target temperature (298 K/25°C) and afterwards equilibrated for 1 min before starting the locking and shimming procedure followed by data acquisition. Sample handling at room temperature and/or acquisition temperature (25°C) was limited to a maximum period of 30 min per measurement to reduce sample degradation. DSS was used as an internal chemical shift reference.

Raw data were processed by a standardized automated protocol using an exponential function with a line-broadening factor of 0.3 followed by automatic phase and baseline correction. Remaining small phase and baseline distortions of the spectra were corrected manually. 

### 2.6. Single Metabolite Quantification, Analysis, and Evaluation

The quantification of organic metabolites was accomplished by a custom-designed algorithm for line-shape analysis. A set of ^1^H NMR signals characteristic for a specific metabolite was fitted by custom-designed software (numares GROUP) and the peak area was determined by integration. With this software, the signals were fitted to a Lorentz function using the position and the width of the signal, as well as the spectral region in which a corresponding signal is given, if applicable. For citrate and urea all signals were fitted (citrate: two doublets at ~2.65 and ~2.53 ppm; urea: singlet at ~5.78 ppm). For the other metabolites, selected signals were used for quantification: for creatinine the singlet at ~4.05 ppm, for lactate the doublet at ~1.32 ppm, for glucose the doublet at ~5.22 ppm and for PAG the singlet at ~7.42 ppm. The total concentration was then calculated using the calibration data obtained from the defined calibration samples of known concentration measured beforehand. Possible errors due to automatic processing of peak recognition were avoided by manual control of the peaks subjected to quantification. Manual correction was done in less than 10% of all quantifications.

The lower limit of quantification (LLOQ) for single metabolite quantification in urine samples was 10 *μ*g/mL for citrate, lactate, urea, PAG and creatinine and 50 *μ*g/mL for glucose. The LLOQ was set for each parameter using the lowest concentration selected for the calibration curve, with the exception of glucose. In this case the LLOQ was limited by the specific lineshape of the ^1^H NMR signal that differs notably from the standard Lorentzian peak shape, thereby complicating the automatic peak recognition and the signal position. For all metabolites, the upper limit of detection (LOD) was 10,000 *μ*g/mL due to the highest concentration used in the calibration curve.

Different measures (mean values, SD, coefficient of determination (*R* ^2^), root-mean-square error (RMSE) and coefficient of variance (CV)) were calculated for specific groups of samples and/or measurements depending on the goal of the analysis as indicated in the respective results paragraph, tables, and/or figures. For better comparability of the effects studied, relative concentrations were calculated for some of the figures as indicated. 

### 2.7. Multivariate Analysis of ^1^H NMR Spectra (Metabonomics Approach)

Prediction of drug induced nephrotoxicity regarding the proximal tubulus was pursued by an ensemble of local experts trained using a set of urine ^1^H NMR data of rats treated with reference compounds [19]. The ensemble classification approach is based on the training of a set of local experts and final classification by an optimized selection of the experts' predictions [20]. To do this, spectra were initially scaled to an equal integral of 1000 units and binned [21] using a bucket width of 0.001 ppm. Spectral regions of interest (SROIs) were selected by a sliding window approach and aligned by a principle component analysis (PCA)-based alignment procedure for compensation of peak shifts [own unpublished data]. Classification of SROIs was achieved by support vector machines (SVMs) [22] using a radial basis function kernel, and final prediction is achieved by majority voting of an optimized set of local experts. The percentage of experts in the ensemble voting for classification of a compound as “toxic” can serve as an indicator of the degree of induced organ toxicity, whereby a percentage over 50% generally leads to classification as “toxic”. 

## 3. Results 

### 3.1. Qualification of ^1^H NMR Quantification

**Figure 1:**
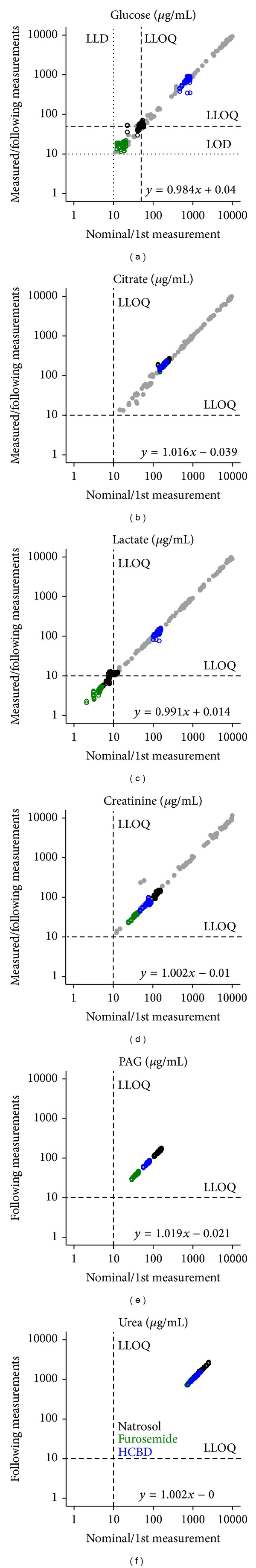
Evaluation of metabolite quantification. Two data sets, synthetic and urine samples, are plotted in this figure to give a measure for accuracy and precision, respectively: (1) nominal and measured concentrations for synthetic samples were measured to determine accuracy and (2) data of first and repeated measurements of urine samples were compared to determine precision. The synthetic samples (grey) were composed of a set of 4 metabolites (glucose, citrate, lactate, creatinine) in aqueous solution at arbitrarily varying concentrations ranging from 10 to 10,000 *μ*g/mL (*n* = 100, glucose: *n* = 89). The nominal metabolite concentration is plotted against the metabolite concentration measured with 1D ^1^H NMR spectroscopy. Urine samples from rats treated with natrosol (black), furosemide (green), or HCBD (blue) with or without pH or salt modifications (“stability test” dataset) were measured repeatedly. Eight repeated measurements per sample (performed over 14 days with refrigerating at about 4°C) are plotted against the first measurement to show reproducibility (*n* = 54 except for glucose: *n* = 47, other values are missing since they were below LOD; 3 treatments ∗ 18 modifications (“original” + 9 pH changes + 8 salt modifications) = 54, for definition of pooled samples see Section 2.4). Equations for linear regression curves are given for all data fromsynthetic and urine samples, which were above LLOQ nominally/with first themeasurement. Dashed lines represent the LLOQ, dotted lines thee LOD (glucose only).

As the first step towards technical qualification, different measures for accuracy of metabolite quantification were calculated using a set of 100 synthetic samples spiked with four selected metabolites: citrate, lactate, glucose, and creatinine in concentrations ranging from 10 to 10,000 *μ*g/mL (Figure 1, grey dots). These data were supported by a set of rat urine samples, where PAG and urea were quantified in addition to the four metabolites (Figure 1, coloured dots). The limits of quantification were taken from the highest and lowest calibration samples, that is, 10 to 10,000 *μ*g/mL (except for glucose; see Section 2.3), which roughly reflected the expected metabolite concentrations in urine [16, 17] (Supplement Figure 1). 

Nominal and measured concentrations for the four or six metabolites, respectively, fitted in well (Figure 1) with *R* ^2^ values ranging from 0.988 (urea) to 0.998 (lactate) and RMSE of not more than 0.05 (Table 2) when considering only those samples with nominal concentrations above the LLOQ. Although quantification was manually controlled, outliers could not be avoided completely. The reason for this was the varying concentrations of citrate in the synthetic samples, leading to changes in the pH values, which then result in variations in the chemical shift. Large variations of the chemical shift may push the specific metabolite signal outside the spectral region in which the signal is searched, consequently leading to a false quantification. 

Further, to determine the reproducibility of NMR experiments, the coefficient of variation (CV) was calculated for the urine samples. In the case of samples with first measured concentrations well above the LLOQ, good reproducibility was achieved with CV values in the range of 2.1% to 3.5% (Table 2). Glucose and lactate (values at or even below LLOQ) achieved acceptable CV values of 6.9% and 4.9%, respectively. These are thus far below the recommendations from the “Guidance for industry” [12], suggesting CV values generally <±15% and near LLOQ <±20%.

### 3.2. Effects of Drug Treatment

**Figure 2:**
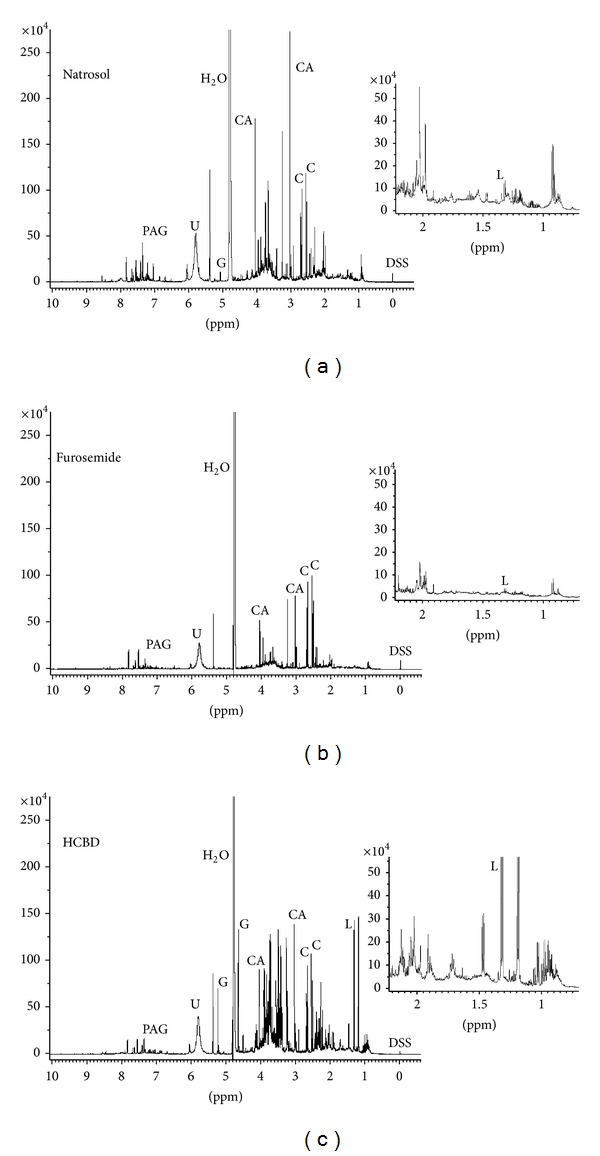
^1^H NMR spectra of rat urine after treatment with natrosol, furosemide, or HCBD. Rats were treated with natrosol, furosemide, or HCBD and urine was collected at 0–8 h for furosemide and at 8–24 h for natrosol and HCBD to achieve pharmacological effects. Typical 1D ^1^H NMR NOESY spectra are shown from rat urine after treatment. All spectra were recorded under standard conditions for single metabolite quantification and plotted with an identical scale. Abbreviations: C: citrate; CA: creatinine; DSS: 4,4-dimethyl-4-silapentane-1-sulfonic acid; G: glucose; L: lactate; U: urea.

Treatment of rats with the diuretic furosemide increases urine volume and salt excretion compared to treatment with the vehicle natrosol, thus diluting urine metabolites. This is seen as a reduction of signal intensity in all peaks observed (Figure 2), and became most prominent for the peaks of urea (5.7 ppm) and creatinine (3 ppm). Further, a lower (i.e., diluted) protein background was observed in the form of a decrease in amino acid signals (CH at 1-2 ppm and NH at 6–7.5 ppm). Quantification of selected metabolites showed lower concentrations for samples from furosemide-treated rats compared to natrosol-treated animals for glucose, lactate, creatinine and PAG, and slightly for urea, while no relevant differences were seen for citrate (Figure 1). 

Treatment of rats with the nephrotoxicant HCBD changed the ^1^H NMR pattern of the urine sample even more (Figure 2). It increased glucose (4.7 ppm and 5.3 ppm), lactate (1.3 ppm), and protein signals (e.g., between 3 and 4 ppm; [23, 24]). This visual impression (Figure 2) is in agreement with quantification of selected metabolites (Figure 1), showing large increases for glucose and lactate, and slight decreases for creatinine, PAG, and even less for urea, while citrate was again not affected. 

### 3.3. Effects from pH and Salt Variations

We next investigated effects from changes in pH and salt concentration on the quantification of metabolites in order to determine possible measurement artefacts. We therefore changed pH and salt concentrations artificially by adding acid, base, or concentrated NaCl solution to the urine samples from treated rats, within (pH) or even above (salt) the pharmacologically observed range (see Supplement Figure 2). pH achieved a range of 2.90 to 9.15 before and 6.62 to 7.64 after buffering (Supplement Table 1). Sodium concentrations ranged from 17 to 87 mmol/L and chloride concentrations from 45 to 152 mmol/L in the original samples. Salt concentrations of these samples were raised about 20-fold by adding NaCl solution (Supplement Table 2), which was confirmed by measuring the increase in osmolality. 

Urine samples from natrosol-, furosemide-, or HCBD-treated rats were used before and after modification, and six selected metabolites were quantified by ^1^H NMR spectroscopy. Data with metabolite concentrations near or below the LLOQ were excluded, that is, glucose and lactate data from the samples of natrosol- and furosemide-treated rats. Additionally, a metabonomics-based prediction of proximal tubule nephrotoxicity was used to monitor the relevance of possible changes in a multivariate approach.

**Figure 3:**
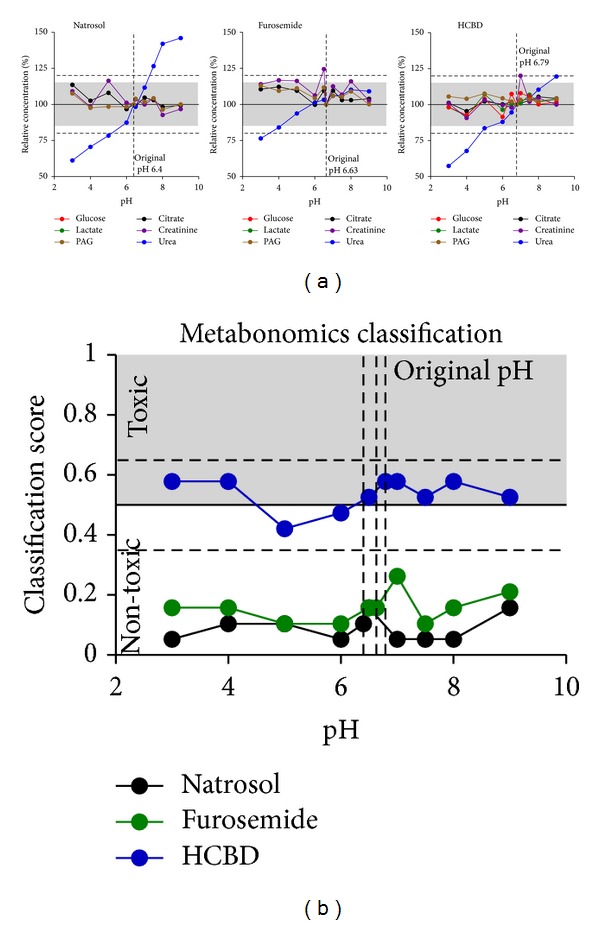
Effects of pH variation. Glucose, citrate, lactate, creatinine, PAG, and urea were measured in urine samples from natrosol-, furosemide-, or HCBD-treated rats by ^1^H NMR metabolite quantification. pH of the original samples varied from ~3 to 9, but much less after addition of phosphate buffer (6.62 to 7.64). Values given on the abscissa are initial pH values measured before addition of phosphate buffer (*n* = 1 per dot from pooled rat urine, for sample definition see Section 2.4). (a) Single metabolite quantification results are given as relative concentrations normalized to the original, not modified sample of each treatment. Values close to or below LLOQ (i.e., glucose and lactate concentrations in the samples from natrosol- and furosemide-treated rats) were excluded from evaluation. The pH values of the respective original samples were plotted as vertical dotted lines. The horizontal grey range (15%) and dotted horizontal lines (20%) show the range of variation stated as acceptable by the “Guidance for Industry” [12]. (b) Prediction of nephrotoxicity was performed using a metabonomics approach (ensemble classification system). For each sample a classifier value was given, labelling the given compound as “(non-)toxic”. The horizontal line at 0.5 is the limit for the prediction of nephrotoxicity, the dashed lines at 0.35 and 0.65 represent an “intermediate” range, related to the statistical significance of the prediction.

Artificial variation of pH even with these extreme changes in the range from 3 to 9 followed by buffering of the samples (pH range 6.62 to 7.64) resulted in relatively mild effects on the single metabolite quantification except for urea (Figure 3(a)). The relative changes were typically smaller than ±15–20%, a limit which was recommended by the FDA for a mean of 5 repeated measurements as compared to the theoretical value [12]. No relevant effects were seen on glucose, citrate, lactate and PAG, which all stayed within the 15% limit, and creatinine, which was mostly in the 15% limits, rarely up to 20% variation. In contrast, the only metabolite affected by pH was urea: an almost 50% deviation from the value detected for the original sample was measured especially at lower pH values (Figure 3(a)). The course of the effect differed in-between the treated samples, and only for pH 6 to pH 7 all samples remained within the 15% limit. Additionally, the creatinine value of the furosemide pH 6.5 sample exceeded the 20% threshold. This measured value remained unexplained, but was most probably due to creatinine concentration changes during the handling of the sample, since repeated measures gave repeatedly high values (compare Figure 5(a)). In agreement with only small effects on single metabolites, pH variation also only slightly affected the metabonomics analysis using an ensemble classification system (Figure 3(b)). For this, ^1^H NMR spectra were classified by pattern classification methods to predict proximal tubule nephrotoxicity. Urine samples were associated with a value between 0 and 1, classifying the sample as non-nephrotoxic with values from 0 to 0.5 and as nephrotoxic from 0.5 to 1. Based on the experience with this assay, the prediction should be differentiated between clearly “toxic” (e.g., >0.65), clearly “non-toxic” (e.g., <0.35) and “intermediate” (0.35–0.65), related to the statistical significance of the prediction. Expectedly, original samples from natrosol-and furosemide-treated rats were predicted “non-toxic” (below 0.35), and prediction was not influenced by changes of pH. Urine from HCBD-treated rats was predicted to be a weak nephrotoxicant with a value of 0.58 (subclassified in the “intermediate range”), suggesting a weak potential as a nephrotoxicant after single compound administration, which may become more severe after multiple administrations. Again, within the accepted variation, changes of pH did not influence the prediction of samples from HCBD-treated rats as “intermediate”. Nevertheless, at pH 5 and 6 correct classification as “toxic” was not achieved. Taken together, pH changes in the range of pH 3 to pH 9 did not relevantly influence prediction of nephrotoxicity, because all calculated values did not change the classification as either negative (samples from natrosol- and furosemide-treated rats) or borderline positive (“intermediate”, samples from HCBD-treated rats).

**Figure 4:**
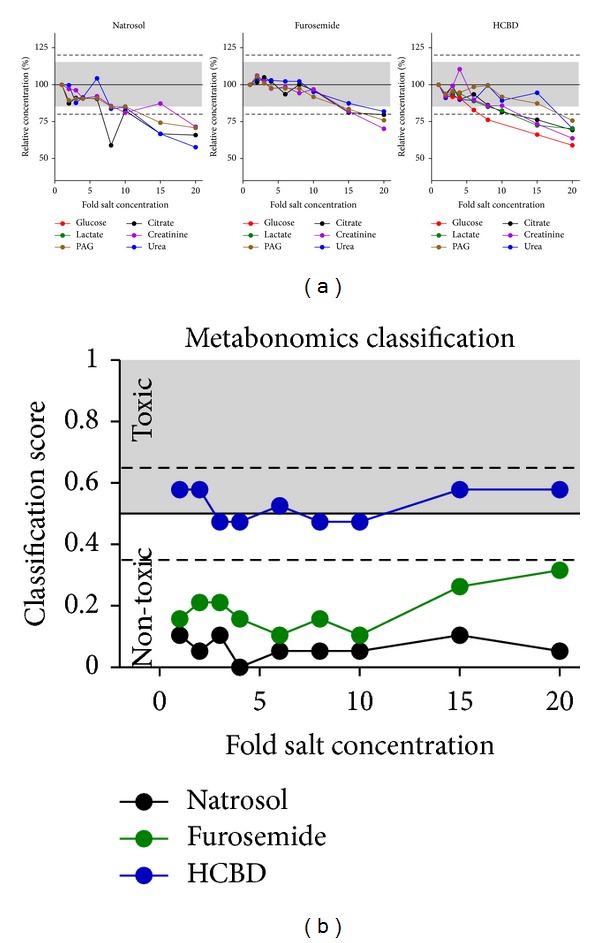
Effects of salt concentration increase. Glucose, citrate, lactate, creatinine, PAG, and urea were measured in urine samples from natrosol-, furosemide-, or HCBD-treated rats by ^1^H NMR metabolite quantification. Values given on the abscissa are relative salt concentrations adjusted by adding 35% NaCl solution to the original sample (see Supplement Table 2) (*n* = 1 per dot from pooled rat urine, for sample definition see Section 2.4). (a) Single metabolite quantification results are given as relative concentrations normalized to the original, not modified sample of each treatment. Values close to or below LLOQ (i.e., glucose and lactate concentrations in the samples from natrosol-and furosemide-treated rats) were excluded from evaluation. The grey range (15%) and dotted horizontal lines (20%) show the range of variation stated as acceptable by the “Guidance for Industry” [12]. (b) Prediction of nephrotoxicity was performed using a metabonomics approach (ensemble classification system). For each sample a classifier value is given, labelling the given compound as “(non-)toxic”. The horizontal line at 0.5 is the limit for the prediction of nephrotoxicity, the dashed lines at 0.35 and 0.65 represent an “intermediate” range, related to the statistical significance of the prediction.

**Figure 5:**
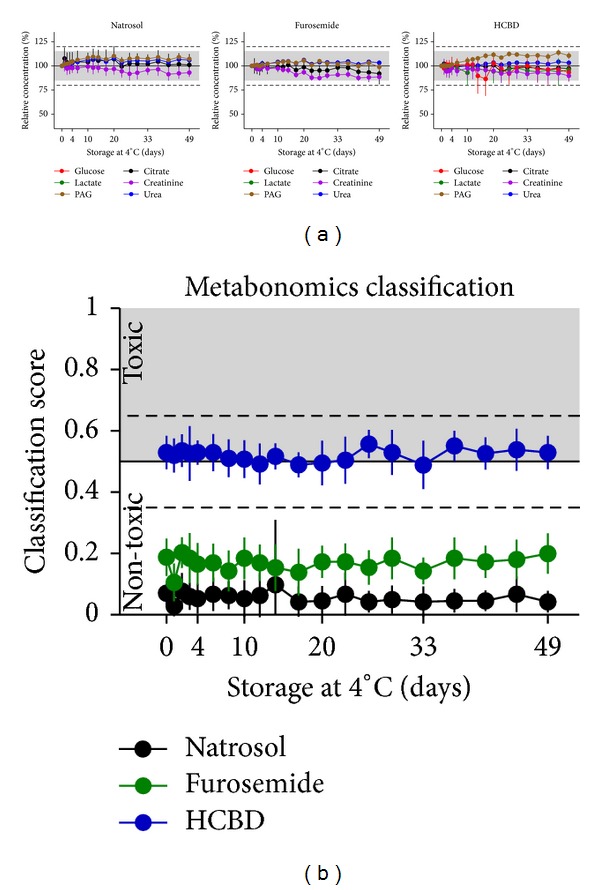
Effects of storage at 4°C. Glucose, citrate, lactate, creatinine, PAG, and urea were measured in urine samples from natrosol-, furosemide-, or HCBD-treated rats by ^1^H NMR metabolite quantification. Repeated measures after storage in the refridgerator at about 4°C were done for the first 5 days at daily intervals, then at longer intervals over a total of 49 days after sample preparation (mean ± SD for *n* = 14–17 per treatment group, including all samples with modified pH or salt concentration, for sample definition see Section 2.4). Samples with suggested microbial degradation were excluded from this evaluation in order to determine only technical variance. (a) Single metabolite quantification results are given as relative concentrations normalized to the first measurement of the respective modified sample. Values close to or below LLOQ (i.e., glucose and lactate concentrations in the samples from natrosol- and furosemide-treated rats) were excluded from evaluation. The grey range (15%) and dotted horizontal lines (20%) show the range of variation stated as acceptable by the “Guidance for Industry” [12]. (b) Prediction of nephrotoxicity was performed using a metabonomics approach (ensemble classification system). For each sample a classifier value is given, labelling the given compound as “(non-)toxic”. The horizontal line at 0.5 is the limit for the prediction of nephrotoxicity, the dashed lines at 0.35 and 0.65 represent an “intermediate” range, related to the statistical significance of the prediction.

The effects of salinity on reproducibility of measurements are depicted in Figure 4. The rise in salinity generally led to a loss in the metabolite concentrations detected. This effect is associated with a typical line-broadening effect in the ^1^H NMR spectra (data not shown). The recommended limit of 15% was exceeded at the earliest at an 8-fold increase in salinity for all metabolites except for glucose, which was already influenced at a 6-fold increase in salinity by 17%. Urea values were—in contrast to effects from pH changes—stable with increasing salt concentrations, as observed in all three treatment groups; effects of more than 20% were seen at the earliest with 15-fold increase of salinity. Effects on compound predictions as nephrotoxicant using the metabonomics classification (Figure 5(b)) were more robust than single metabolite measurements: no relevant change of prediction was observed for samples from natrosol-, furosemide-, and HCBD-treated rats. The only slight modification was seen for samples after furosemide treatment, which showed an increase in absolute values from ~0.2 to 0.32 starting at 15-fold salt increases. 

### 3.4. Effects of Sample Storage

**Figure 6:**
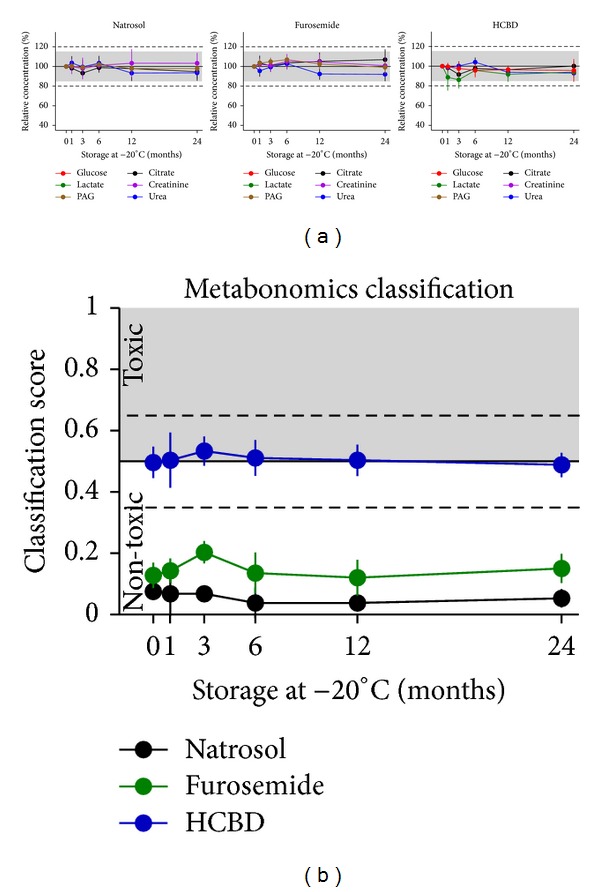
Effects of storage at −20°C. Glucose, citrate, lactate, creatinine, PAG, and urea were measured in urine samples from natrosol-, furosemide-, or HCBD-treated rats by ^1^H NMR metabolite quantification. Samples were stored at –20°C over a period of up to 24 months; for each time-point a separate aliquot was used (mean ± SD for *n* = 7 per treatment group, including a selected set of samples with modified pH or salt concentration, for sample definition see Section 2.4). Samples were thawed by a standardized procedure shortly before measurement. (a) Single metabolite quantification results are given as relative concentrations normalized to the first measurement of the respective modified sample. Values close to or below LLOQ (i.e., glucose and lactate concentrations in the samples from natrosol- and furosemide-treated rats) excluded from evaluation. The grey range (15%) and dotted horizontal lines (20%) show the range of variation stated as acceptable by the “Guidance for Industry” [12]. (b) Prediction of nephrotoxicity was performed using a metabonomics approach (ensemble classification system). For each sample a classifier value is given, labelling the given compound as “(non-)toxic”. The horizontal line at 0.5 is the limit for the prediction of nephrotoxicity, the dashed lines at 0.35 and 0.65 represent an “intermediate” range, related to the statistical significance of the prediction.

Sample stability is influenced by the quality of sampling, storage time and storage conditions, such as temperature. We thus investigated the effect of storage at 4°C and at −20°C on NMR analysis of urine samples with respect to single metabolite classification (Figures 5(a) and 6(a)) as well as using the metabonomics classification (Figures 5(b) and 6(b)). Again, samples with values close to or below the LLOQ, that is, glucose and lactate in the samples from natrosol- and furosemide-treated rats, were excluded from evaluation.

**Figure 7:**
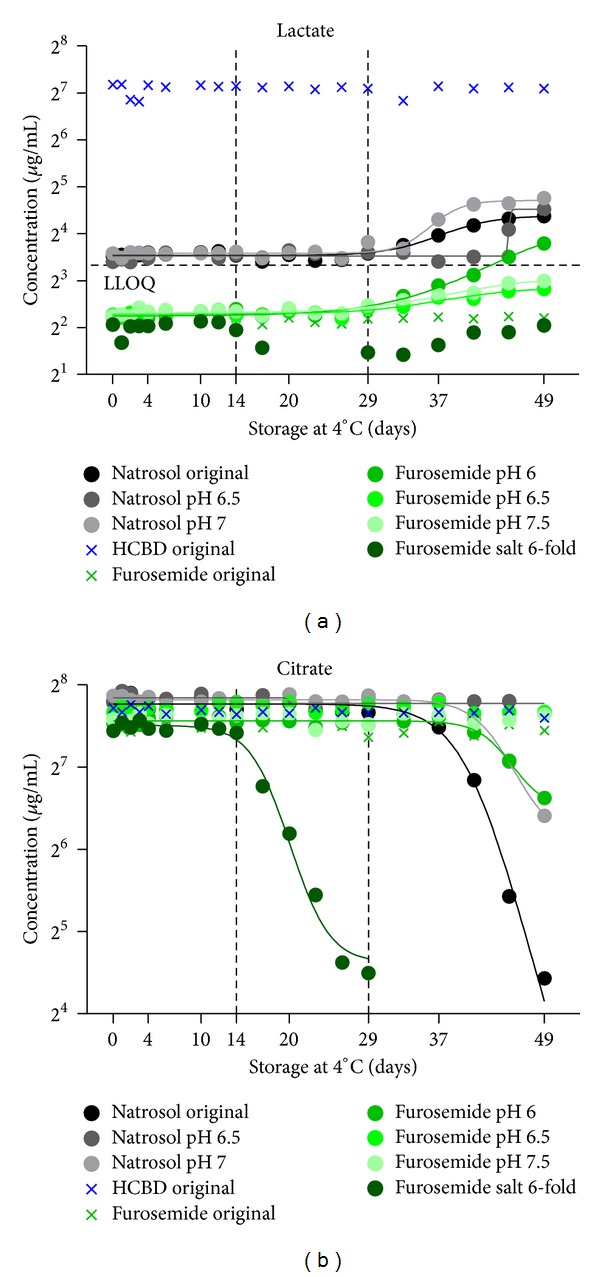
Effects of microbial contamination. Citrate and lactate were measured in urine samples from natrosol-, furosemide-, or HCBD-treated rats by ^1^H NMR metabolite quantification with or without modifications of pH or salt (stability test dataset, *n* = 1). Samples were measured during storage at 4°C over a period of 49 days. A selection of samples showing typical effects on citrate and lactate from possible microbial degradation processes is plotted (circle) in comparison with not contaminated control samples (cross), and fitted with a non-linear curve fit. The colour of the symbols indicates animal treatment (natrosol, furosemide, and HCBD).

Even though samples were collected as clean as possible, changes in lactate and citrate were identified in the refrigerated samples, which suggest microbial contamination (Figure 7). Comparable changes in metabolite patterns from microbial growth were described previously [2, 25]. Changes in lactate and citrate were observed in 8 of 54 (15%) of the samples, most of them with near physiological pH. The observations started at the earliest on day 14 (drop of citrate) and on day 29 (increase in lactate). Relative changes until day 14 did not exceed the 15% limit for citrate. For lactate, samples above LLOQ were mostly within the 15% range, and only the sample after HCBD treatment was twice within the 20% range (original and pH 6.5 modification), and once even reduced by 22% on day 3.

Analysis of refrigerating effects at 4°C was done after exclusion of samples with suggested microbial contamination. Refrigerating of urine samples up to 49 days after a single freezing period at −20°C had no relevant effect on NMR analysis (Figure 5(a)), that is, the absolute concentrations of the measured metabolites remained well within the ±15% range for means from repetitive measures suggested in the “Guidance for Industry” [12] for all six measured metabolites in all three types of samples. Also with the metabonomics classification, results were not affected by storage at 4°C over the period in question (Figure 5(b)). 

Effects of freezing at −20°C were monitored over two years (Figure 6) using separate aliquots of samples of the stability test. Measurements for the six single metabolites were not relevantly influenced over two years, that is, all values remained within the ±15% CV limits (Figure 6(a)). In line with single metabolite measures, the metabonomics classification was also not relevantly affected when storage was done even for two years at −20°C (Figure 6(b)). 

**Figure 8:**
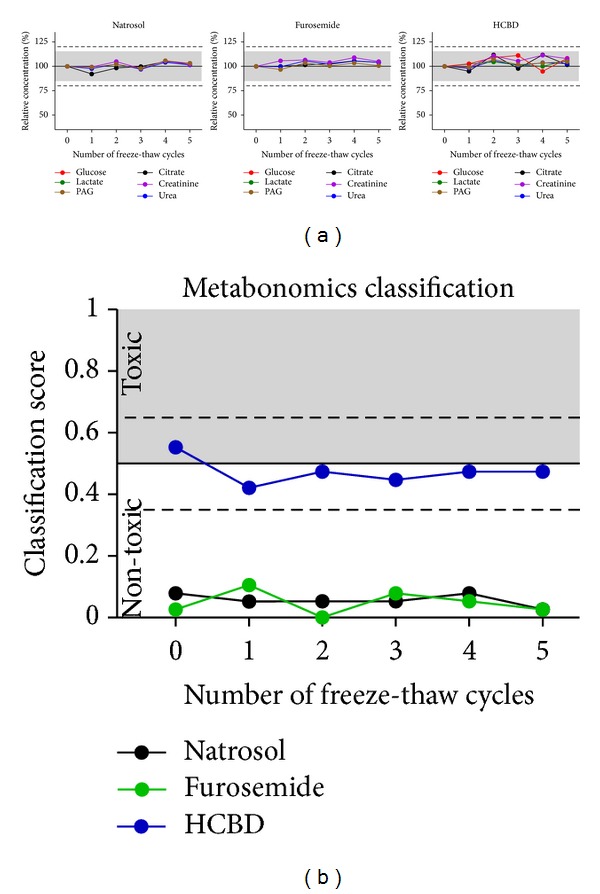
Effects of multiple freeze-thaw cycles. Glucose, citrate, lactate, creatinine, PAG, and urea were measured in urine samples from natrosol-, furosemide-, or HCBD-treated rats by ^1^H NMR quantification. Samples were stored at −20°C before and in-between the measurements (*n* = 1 per dot, urine samples pooled treatment-wise from two rats). They were thawed by a standardized procedure shortly before the measurements; the procedure was repeated for 5 freeze-thaw cycles. (a) Single metabolite quantification results are given as relative concentrations normalized to the first measurement. Values below LLOQ (i.e., glucose and lactate concentrations in the samples from natrosol- and furosemide-treated rats) were excluded from evaluation. The grey range (15%) and dotted horizontal lines (20%) show the range of variation stated as acceptable by the “Guidance for Industry” [12]. (b) Prediction of nephrotoxicity was performed using a metabonomics approach (ensemble classification system). For each sample a classifier value is given, labelling the given compound as “(non-)toxic”. The horizontal line at 0.5 is the limit for the prediction of nephrotoxicity, the dashed lines at 0.35 and 0.65 represent an “intermediate” range, related to the statistical significance of the prediction.

Investigation of possible artefacts from multiple freeze-thaw cycles was done over 5 freeze-thaw cycles. No relevant changes of single metabolite concentrations (Figure 8(a)) or the metabonomics classification (Figure 8(b)) were observed. Since a different set of urine samples was used for the investigation of freeze-thaw cycles (Figure 8(b)) as compared to the refrigerating (Figure 5(a)) and freezing (Figure 6(a)) effects, absolute values in the metabonomics classification were slightly different. Nevertheless, general classification was very well comparable, again supporting reproducibility of measurement and classification techniques.

### 3.5. Biological Variance

Biological and technical variance was compared in order to evaluate whether technical reproducibility may affect the interpretation of results from biological experiments. We thus plotted both sources of variability in one plot for citrate, creatinine, PAG and urea (Figure 3), while glucose and lactate were not depicted since most values were below LLOQ. 

**Figure 9:**
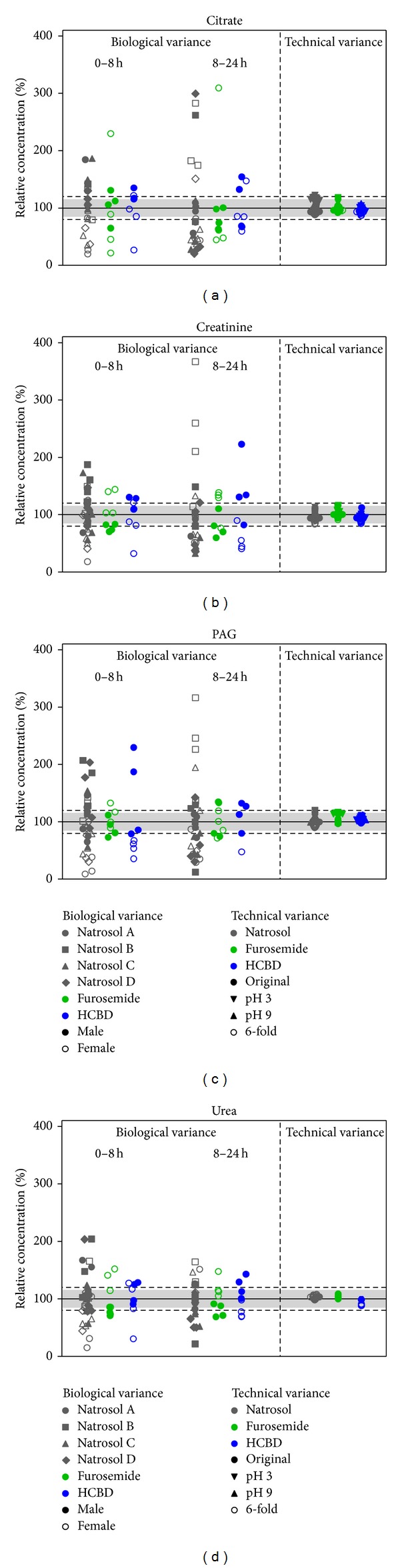
Comparison of biological and technical variance. Citrate, creatinine, PAG, and urea were measured in urine samples from natrosol-, furosemide-, or HCBD-treated rats by ^1^H NMR metabolite quantification. Glucose and lactate values were close to or below the LLOQ and thus excluded from evaluation. To depict biological variance, samples from individual animals (*n* = 8 per treatment group, one dot represents one individual animal), including 4 male (filled symbol) and 4 female (open symbol) animals, were measured. Data from natrosol-treated animals are available from 4 different treatment days ((a), (b), (c), (d); different animals were used on different treatment days). Technical variance is determined from pooled rat urine samples, which are additionally modified concerning pH (depicted values for pH 3 and 9 except for urea) and 6-fold increases in salt-concentration, that is, changes in the accepted range (see before). Additionally, each sample is measured 9 times up to day 14. Values are given as relative concentrations normalized to the mean of the respective treatment and period (biological variance) or the value from the first measurement of the original sample (technical variance). The horizontal grey range (15%) and dotted horizontal lines (20%) show the range of variation stated as acceptable by the “Guidance for Industry” [12].

Biological variance is driven by various factors; general interindividual variance may be enhanced further by day-to-day variance or diurnal changes and gender effects. To reflect this, we used data from male and female rats, different collection periods, three treatment groups as well as data from different treatment days (Figure 9). Normalizing these data to each subgroup mean showed that technical variance (<15%) was relevantly lower than biological variance, which was typically in the range of 1.5- to 2-fold changes, and up to nearly 3-fold changes in a low number of animals. Females tended to discriminate from males at certain points of time or for selected parameters, for example, lower citrate, creatinine, PAG and urea values in the 0–8 h natrosol-treated group, but patterns changed after different treatments or in other collection periods. The points of time after treatment or the treatment itself led to relevant absolute changes (Figure 1), but did not relevantly change the variability as interpreted from the degree of spreading (Figure 9). Even different treatment days did not form relevant subgroups (see natrosol). Thus, biological variance is mainly driven by interindividual variance and is by far larger than technical variance.

## 4. Discussion 

### 4.1. Reasons for Selection of Method

If not chosen carefully, the measuring technique may influence the outcome of an experiment substantially by possibly leading to unconsidered artefacts. Optimal techniques exist for measurements in different sample types of varying sample composition (resulting in variable background noise) and metabolite concentrations. ^1^H NMR spectroscopy is a robust measuring technique that detects small molecules with at least one H-atom. Thus, many different molecules can be measured on a single run, and the sample is not destroyed after the measurement. A great advantage of ^1^H NMR spectroscopy is the broad range of concentrations covered by the method (typically about 4 to 5 log ranges). Its sensitivity can be affected by the duration of the measurement, so the ratio of measurement speed to sensitivity should be optimized. To achieve a reasonably high throughput method for use in pharmacological assays of rat urine the LLOQ was set below the range of metabolite concentrations described previously [16, 17] and measured in our own rat urine samples (Supplement Figure 1). ^1^H NMR spectroscopy covered our need for quantification of multiple metabolites for classification of rat urines after treatment with different (reference) compounds using a metabonomics approach to obtain high predictive power. Those metabolites, which are either not within the optimal range of detection, or which are influenced by other experimental factors, such as urea by pH, are not expected to be used in our model due to the selection of SROIs [19]. We thus expect that this approach included a sufficient number of SROIs within the spectra, which are not influenced by any experimental factors, to build a predictive model. However, the chemical structure of the metabolites covered by the SROIs remains unknown. Therefore, we did not aim at having all metabolites within their optimal concentration range, but to have a quick, robust, and cost-effective delivery of sufficient data to support the modelling process. 

### 4.2. Accuracy of Detection and Sensitivity

To evaluate the accuracy of measurement a set of random mixed synthetic samples containing glucose, citrate, creatinine and urea was used. Data were complemented by repeated measurements of rat urine samples, and *R*² and RMSE were used as the read-out. Additionally, for the repeated measurements from rat urine samples, CV values were calculated to investigate reproducibility of the data. 

Accuracy was excellent for all three measures, as shown by *R*²>0.98 and RMSE < 0.05 and CV values ≤3.5% for parameters above LLOQ (Table 2). When including data close to or even below LLOQ (glucose and lactate), CV values increased up to 6.9%, which is still in a well acceptable range. Hence, the technical variance determined in our own experimental setup is similar to or even lower than that determined in other studies with a similar ^1^H NMR-based setup, where *R*² values of 0.984, 0.974 and 0.893 to were calculated for citrate, taurine, and hippurate, respectively, and CV values were below 4.6% also for very low concentrations [26]. Further, our method was superior to the recommendations from the FDA [12]. This is supported by visual inspection of the data: regression curves for the synthetic samples showed that the measured values were exactly those, which were expected; that is, lines went through the origin and had a slope of ~1. This indicates that measures were precise for the quantification of absolute values. For the rat urines, concentrations measured with ^1^H NMR quantification tended to be lower when compared to clinical chemistry data for glucose and urea, and matched for creatinine (Figure 1 compared to Supplement Figure 1). Guidelines for clinical chemistry measurements allow relative deviations for single measurements/RMSE values in urine in the range of 6.5% (sodium) to 15% (albumin); values for glucose, creatinine, and urea are 11%, 12%, and 13.5%, respectively, and thus much larger than the values found using ^1^H NMR quantification [27]. These values are given for a range of factor 60 (glucose) to 300 (creatinine) [27], which is much smaller than the ~4 log units covered by ^1^H NMR quantification. Thus with our setting, ^1^H NMR quantification of metabolites is more accurate in a longer linear range compared to quantification with clinical chemistry. When compared to other methods for quantifying multiple metabolites, it was observed that mass spectrometry (MS) is more sensitive than NMR [28], but NMR is more suitable for metabolite quantification than MS [29]. Antibody-based quantification of single metabolites (with or without multiplexing technology), such as ELISA, Mesoscale or Luminex technologies have often only a small linear range, which is stated to be best for the mesoscale technology. Nevertheless, these methods are often influenced by matrix effects, not allowing different dilution steps. CV values for different assays are published for the Luminex technology and range from <5% to >20%, with most assays in the range from 5% to 10% [30]. Thus, CV values from the Luminex technology were not better than those of ^1^H NMR-based metabolite quantification, where we found most values <5% and largest values <10% (Table 2). 

Sensitivity of the chosen measurement protocol was sufficient to measure most of the rat urine samples: only concentrations for glucose and lactate were near or below the LLOQ, especially after treatment with furosemide (Figure 1). For all metabolites except glucose, the LLOQ was set by the preselected concentrations of the calibration samples, and their signals were still well above the background noise of the ^1^H NMR signal. Thus, the sensitivity of the assay can easily be enhanced by adding a lower calibration sample concentration. Only for glucose was sensitivity limited by the typical line shape and position of the glucose signal in the ^1^H NMR spectra to 50 *μ*g/mL. If measurements of lower concentrations or higher accuracy in the low range were needed for glucose, this could be reached by enhancing measurement cycles/times and by increasing spectral resolution (in addition to adding a lower calibration sample concentration). Nevertheless, the detection of pathological events, such as the increase of glucose concentration after HCBD treatment (Figure 1), is easily possible with this setup. 

Thus, even with the short measurement times used here for screening applications, very high accuracy of detection was achieved. For specific applications the use of (additional) high resolution ^1^H NMR spectra could further enhance sensitivity as well as accuracy [31], but thereby increase measurement time. 

### 4.3. Buffering Conditions, Effects of pH Variation and of Increased Salt Concentration

In general, sample changes in pH and salt can affect ^1^H NMR quantification, but the magnitude depends on the type of sample. While blood (serum/plasma) is highly regulated *in vivo* and thus samples typically do not vary much, composition of urine samples may vary markedly, depending on physiological factors, such as nutrition, gender, time of day in addition to pharmacological/pathological factors caused by the kidneys maintaining body homeostasis. Thus, urine pH is highly variable. Rat urine typically covered a pH range from 5.5 to 8.5 (mean ± SD was 6.8 ± 1.1) depending on the time of day and on the metabolic status of the animal, and can reach extreme values after treatment of rats with different pharmaceuticals between pH 4.0 and 9.1 (Supplement Figure 2). Similar ranges were also described for mice, covering an even slightly broader range from 3 to 9 [3], which is supported by own unpublished data showing a pH range in mice from 3.9 to 7.8, with a mean ± SD of 7.2 ± 0.7 (*n* = 32) for NMRI mice and of 5.4 ± 0.7 (*n* = 77) for a different breed, showing in addition breed-specific normal ranges. In men, including healthy volunteers and patients with different treatments and for different illnesses, we found a pH range from 3.0 to 8.7 with a mean ± SD of 5.1 ± 1.3 (*n* = 265 samples) in treated patients and of 5.0 ± 1.2 in healthy volunteers (*n* = 104 samples). This indicated similar wide pH ranges observed in different species including men.

Changes in H protonation interfere with quantification by ^1^H NMR spectroscopy, especially since variation of pH induces changes in the exact peak positions, which may influence metabolite quantification. Thus, buffering of the samples is essential to achieve low variation of pH in the samples finally measured. We chose a phosphate buffering system to set pH of the sample to 7.4 based on prior testing and in accordance with other published buffering methods [4, 31]. Buffers using buffer systems other than phosphate [1] were not considered either due to their buffering pH range or due to the introduction of compounds producing high intensity signals in the sample (and thus interfering with metabolite quantification). Buffering capacity was found to be sufficient at a concentration of 266 mM: the initial sample pH of 2.90 to 9.15 was thereby reduced to a range of 6.62 to 7.64 (Supplement Table 1) and no precipitation was observed in the samples. Higher final concentrations of the buffer up to 1 M have been proposed by Lauridsen for concentrated urine [2]. We observed precipitation when using higher buffer concentrations up to 333 mM (own unpublished data) and further, such high buffer concentrations contribute relevantly to sample salinity, which was identified to be a very critical parameter for quantification of metabolites at these levels (see below). 

Furthermore, all metabolites except for urea were not relevantly influenced by changes of pH (Figure 3(a)), and the metabonomics classification approach resulted in comparable results for the whole pH range (Figure 3(b)). Obvious changes were only seen for urea (low and high pH). Urea is known to be sensitive to pH changes in the range of pH 6.62 to 7.64 due to its pK value such that urea exchanges protons with the solvent leading to NMR exchange broadening. And since line broadening influences the quality of the fit of the corresponding signal, these effects have been expected. We thus recommend quantifying urea only in a sample pH range from 6 to 7. In summary, in a sample pH range from 3 to 9, no relevant effects are to be expected for metabolites with pK values not in the planned buffering range of sample pH. In contrast, for metabolites with pK values in the planned buffering range of sample pH like urea, effects of pH need to be tested before starting metabolite quantification. 

Salt composition of samples with biological origin may vary, especially in the case of urine samples, because the kidney regulates salt homeostasis. Typical rat urine ion concentrations of Na^+^ and Cl^−^ are ~20 mmol/L and ~30 mmol/L, respectively (Supplement Figure 2). Pharmacological effects for example, by diuretics can enhance this up to ≥100 mmol/L or by antidiuretics or urine diluents down to ≤10 mmol/L (Supplement Figure 2). Typically, osmolality in rat urine is ~300 mosmol/L and may increase further up to ≥600 mosmol/L, with maximal measured values of nearly 1300 mosmol/L (Supplement Figure 2). Thus, the selected artificial increase of osmolality by salt addition up to 1900 mosmol/L (Supplement Table 2) is relevantly stronger than typical pharmacological effects. 

Depending on the chemical structure of the metabolite, salinity can cause line broadening of the corresponding NMR signal, thereby leading to differences between measured and nominal concentrations. Effects of salt addition were stronger in the samples after treatment with natrosol and HCBD than after furosemide (Figure 4(a)); this effect is correlated with initial sample osmolality (Supplement Table 2). Based on the single metabolite quantifications, a 6-fold increase in salinity was chosen as a conservative limit for increases in salt concentrations (Figure 4(a)), provided measurements are performed above LLOQ. This is mainly driven by the decrease seen for glucose quantification, starting already at the 6-fold increase in salinity, while the other metabolites started mostly at higher increases in salinity. This 6-fold increase in salinity was associated with a measured osmolality of ~700–900 mosmol/L (Supplement Table 2) and thus near maximal measured osmolality in rat urines after treatment (Supplement Figure 2). When increases of salinity were about 10-fold (i.e., osmolality of ~900–1200 mosm/L, Supplement Table 2), salt-induced changes in quantification were significantly larger than 15% for several metabolites (Figure 4(a)), and were thus out of the acceptable range [12]. Despite this, salinity increases even up to 10-fold did not lead to relevant changes in the results of the metabonomics classification approach, and only at 15-fold increases in salt concentration the classification value rose for the furosemide-treated group (Figure 4(b)). Since these salt concentrations were well above those of normal or even pathological urine samples, ^1^H NMR analysis of urine samples with pathological variation of salt-concentrations is feasible with high accuracy. Nevertheless, for samples with expected severely increased salinity, a routine determination of salt concentrations or osmolality would be useful as a quality check before single metabolite quantification by ^1^H NMR. 

In metabonomic studies, peak shifts from variability of pH or salinity are generally addressed as a problem [32], especially when unsupervised methods are used due to the large amounts of data. We applied an automated alignment procedure to compensate such variation in the exact position of peak signals. With that procedure, prediction of nephrotoxicity was always correct for natrosol and furosemide as “non-toxic” compounds, and for HCBD as a potential weak nephrotoxicant with “intermediate” classification (Figures 3(b) and 4(b)). Thus, semi-automized quantification of samples varying in pH and salinity can even be used for metabonomics classification of data, when appropriate buffering conditions are chosen. 

It was previously reported that effects of pH and salt on ^1^H NMR measurements can be overcome by different evaluation techniques. This was described by Mercier et al. successfully for serum and cerebral fluid, while they did not overcome the difficulties from urine [33]. In contrast, Veselkov et al. used a peak alignment method [34] and Jiang et al. specific buffering systems [11] to minimize these effects in urine. This is in agreement with our data, showing that physiological and pathological changes in rat urine can be compensated by well-chosen buffering techniques and optimized evaluation techniques, resulting in an excellent reproducibility of quantification of metabolites as well as of a well acceptable reproducibility of classification using a metabonomics approach as a marker for relevance of these changes.

### 4.4. Effects of Sample Storage at Refrigerated and Frozen Conditions including Microbial Effects

During storage, sample composition can be changed by chemical and microbial metabolite degradation. Standard methods to delay degradation are mostly freezing at −20°C or −80°C. Refrigerating at 4°C is less common, but essential for selected determinations such as quantification of enzyme activity using clinical chemistry methods. Degradation may also be influenced by sample handling, especially hygiene standards. Our urine samples were already refrigerated during collection in the metabolic cages. Thereby, we reduced microbial growth, and we avoided the non-standardizable addition of preservatives during collection, such as varying ratios of preservative and urine volume. Moreover, uncontrolled thawing can result in locally increased/decreased salinity during the thawing process, which may lead to precipitation of previously soluble sample components, especially proteins. This can effectively be avoided by using precise sample thawing protocols. 

Under these optimized conditions, samples were stored for at least 14 days under refrigerated conditions without any significant changes in metabolite concentrations, that is, changes remain typically within the 20% limits; thereafter day-to-day variance gradually became larger (Figure 5(a)). Nevertheless, these changes had no influence on the metabonomics classification approach over the whole period of 49 days (Figure 5(b)). In routine laboratory procedures refrigerated storage periods are mostly shorter and thus do not need to be considered as a critical parameter.

The observation of sample composition over a rather long time period of 49 days at 4°C allowed the separation of two different decomposition processes: (a) effects occurring from storage time (e.g., chemical reactions, physical processes) and (b) effects from suggested microbial contamination. Those effects, which are related to storage time, led to slightly reduced reproducibility over time, as indicated by the larger standard deviation in Figure 5(a). It did not result in any relevant changes of measuring absolute concentrations of single metabolites (Figure 5(a)) or in the metabonomics classification approach (Figure 5(b)) over 49 days. This indicates that chemical reactions and physical processes do not relevantly influence the metabolites quantified by us. In about 15% of the samples, an increase of lactate and a decrease of citrate were observed, starting at the earliest at 14 days after sample collection (Figure 7). The typical exponential growth rate and the type of metabolites changed suggests that the effects observed are caused by microbial degradation, most probably by low numbers of bacteria from air and the surfaces of non-sterile sample collection equipment. Most of the samples which were identified as contaminated were original samples or samples with only moderate changes in pH prior to buffering. Since these samples were handled most often and since for example, high salt concentrations typically do not allow bacterial growth, it is assumed that the most critical steps are the non-sterile sample collection and the sample handling. We thus support storage of urine samples up to 14 days, when collected under high hygiene standards. In contrast, Lauridsen et al. did not recommend storage at 4°C without any preservative, since they occasionally observed metabolic changes most probably due to microbial contamination [2]. The use of preservatives is one possibility to minimize microbial growth when longer storage is needed. Sodium azide has no effect on the ^1^H NMR spectra of urine, whereas sodium fluoride causes a shift especially of the citrate resonances [2]; consequently sodium azide should be preferred over sodium fluoride for ^1^H NMR quantification in case the condition of the sample collection cannot be increased to higher standards. 

Freezing at −20°C is the most common and practical method for the storage of samples. We did not see any relevant effects on single metabolite quantification or metabonomics classification up to two years (Figure 6). This is in agreement with data from Lauridsen et al. [2], who evaluated effects of storage, time and temperature, freeze-drying, and the presence of preservatives in human urine. They detected no changes in the ^1^H NMR fingerprints of human urine stored at or below −25°C for 26 weeks. Veljkovic et al. showed that urine may be stored at room temperature at least up to 4 h [6].

We additionally tested whether (multiple) freeze-thaw cycles may have an impact on reproducibility of the measurements. In our study up to 5 freeze-thaw cycles did not interfere with either type of analysis (Figure 8). Thus, samples can be kept frozen or may even be refrozen, which is in agreement with observations from Petri et al. for quantification of proteins in human urine samples by surface-enhanced laser desorption/ionization time-of-flight mass spectrometry (SELDI-TOF-MS) for 8 cycles [35].

Taken together, high standards of sampling quality and standardized sample preparation allow reliable data to be generated even with long term storage or repeated freeze-thaw cycles. Storage of samples at −20°C is the method of choice for ^1^H NMR spectroscopy to ensure optimal data quality, and can be used even for studies with large sample pools or sample collection periods of several months, such as toxicological or clinical studies. 

### 4.5. Relevance of Technical Variability in Comparison to Biological Variability

The statistical power of experiments can be influenced by technical accuracy, as soon as technical variance reaches the magnitude of the expected biological variance. To avoid unnecessary numbers of samples for achieving adequate power, variance of the measurement technique should thus always be well below biological variance.

For biological variance, we tested interindividual variance—a variable expected to be small as compared to effects from pharmaceutics or animal health status. Effects from the used pharmaceutics and from different collection periods were excluded by normalization. Even then, we showed that the biological variance clearly exceeds the technical variance (Figure 9), also in different types of samples, that is, after different treatments of animals, with different gender and at different points of time. Also the artificially induced pH and salt variations did not relevantly affect technical variance as compared to the magnitude of biological variance. Gender differences tended to give trends for certain metabolites at different times or after certain treatments, but still the interindividual changes remained the larger factor. Also the magnitude of day-to-day variance (natrosol-treated groups A, B, C, D) was well below the interindividual changes. This clearly indicates that ^1^H NMR quantification is suitable for routine measurements in (rat) urine, which is in agreement with investigations of Slupsky et al. showing that ^1^H NMR spectroscopy can be used for metabolite quantification in human urine, when algorithms are selected properly [17].

### 4.6. Conclusion

The aim of this study was to analyze the suitability of ^1^H NMR-based quantification of rat urine samples for multivariate data analysis, that is, metabonomics-based classification of the samples. Thereby, we did not aim at having all metabolites within their optimal concentration range, but to have a quick, robust and cost-effective delivery of sufficient data to support the modelling process. We showed that standard methods of (long-term) storage, for example, storage at −20°C, can be used up to two years, and that refrigerating at 4°C could be used up to 14 days. Moreover, artificial pH and salt changes of rat urine samples did not relevantly affect measurement accuracy, thereby showing that exact measurements after only a simple buffering step are feasible. This may best be achieved in a specialized NMR facility for centralized analysis, thereby optimizing the quality of results. This way, ^1^H NMR technology represents a robust, high accuracy, high throughput analytical system for metabolite quantification or for metabonomics classification approaches or for a combined approach for data evaluation. 

## Supplementary Material

The supplementary material provided includes:
(1) A table giving the measured pH values of all stability test samples before and after buffering. The table also includes the dilution factor resulting from the addition of HCl or NaOH.(2) A table giving the salt concentrations and measured osmolality of all stability test samples. The table also includes the dilution factor resulting from the addition of the concentrated NaCl-solution.(3) A histogram depicting glucose, creatinine and urea concentrations in rat urine after treatment of rats with vehicle or reference compounds, i.e. natrosol, furosemide, formoterol, spironolactone, amiloride, acetaminophen, adriamycine or hydrochlorothiazide.(4) A histogram depicting sodium and chloride concentrations, pH and osmolality in rat urine after treatment of rats with vehicle or reference compounds, i.e. natrosol, furosemide, formoterol, spironolactone, amiloride, acetaminophen, adriamycine or hydrochlorothiazide.

## Figures and Tables

**Table 1:** Overview of samples used for the various experiments of this study.

Sample name	Source	Characteristics	Use
Calibration	Synthetic	10, 100, 1,000, 10,000 *µ*g/mL	Calibration for single metabolite quantification
Accuracy test	Synthetic	Defined composition (mixture of 4 metabolites at different concentrations) in a range of 10 to 10,000 *µ*g/mL	Determination of accuracy of measurement
Stability test	Treatment-wise pooled urine samples (urine from 5 male and 5 female rats per pooled sample)	Treatment with natrosol, furosemide, or HCBD	Determination of effects from (i) pH and salinity (ii) Storage (refrigerating, freezing) (iii) Drug treatment
Freeze-thaw test	Treatment-wise pooled urine samples (urine from 2 male rats per pooled sample)	Treatment with natrosol, furosemide, or HCBD	Determination of effects from multiple freeze-thaw cycles
Biological variance test	Urine samples from individual male or female rats	Treatment with natrosol, furosemide, or HCBD	Determination of interindividual biological variance and effects from sample composition on interindividual variance

**Table 2:** Method-related variance.

	Glucose	Citrate	Lactate	Creatinine	PAG	Urea
	Synthetic samples and rat urines (stability test series)					

*R* ^2^	0.994	0.991	0.998	0.992	0.997	0.988
RMSE	0.048	0.038	0.033	0.050	0.021	0.016

	Rat urines (stability test series)					

CV [%]	6.9	3.4	4.9	3.5	3.0	2.1

Synthetic and urine samples as described in Figure 1 were used to calculate different measures of accuracy. *R* ^2^ and RMSE values were calculated for all data from synthetic and urine samples together, which were nominally/with first measure above LLOQ (including 100 synthetic samples and 54 urine samples with 8 repetitions, i.e., maximal *n* = 532). Additionally, CV values were calculated for the repeated measures of the 54 different rat urine samples, each measured in total 9 times. Again, values below LLOQ at the first measure were excluded (but values below LLOQ from repeated measures were included in the evaluation), thereby reducing the number of samples to 25 for lactate and to 24 for glucose. CV values for each metabolite are given as a mean of CV values for the different treatment groups (natrosol, furosemide, or HCBD) and effectors (pH 3 to pH 9, salt up to 20-fold), that is, first calculating the CV values for specific samples, for example, the sample natrosol treatment, pH 3, salt 4-fold, and thereafter calculating mean CV values for each metabolite.

## Abbreviations

C: Citrate
CA: Creatinine
CV: Coefficient of variance
DSS: 4,4-Dimethyl-4-silapentane-1-sulfonic acid
G: Glucose
HCBD: Hexachlorobutadiene
L: Lactate
LOD: Limit of detection
LLOQ: Lower limit of quantification
NMR: Nuclear magnetic resonance
PAG: Phenylacetylglycine
PCA: Principle component analysis
RMSE: Root-mean-square error
SD: Standard deviation
SROI: Spectral region of interest
SVM: Support vector machine
U: Urea.
