# Supplementary material for: Reproducibility of NMR Analysis of Urine Samples: Impact of Sample Preparation, Storage Conditions, and Animal Health Status
Source: Biomed Res Int. 2013 Jun 23;2013:878374. doi: 10.1155/2013/878374 (PMC3705931; doi:10.1155/2013/878374)
Supplement: Supplementary file 1 — The supplementary material provided includes: (1) A table giving the measured pH values of all stability test samples before and after buffering. The table also includes the dilution factor resulting from the addition of HCl or NaOH.(2) A table giving the salt concentrations and measured osmolality of all stability test samples. The table also includes the dilution factor resulting from the addition of the concentrated NaCl-solution.(3) A histogram depicting glucose, creatinine and urea concentrations in rat urine after treatment of rats with vehicle or reference compounds, i.e. natrosol, furosemide, formoterol, spironolactone, amiloride, acetaminophen, adriamycine or hydrochlorothiazide.(4) A histogram depicting sodium and chloride concentrations, pH and osmolality in rat urine after treatment of rats with vehicle or reference compounds, i.e. natrosol, furosemide, formoterol, spironolactone, amiloride, acetaminophen, adriamycine or hydrochlorothiazide. [file 878374.f1.pdf]

Reproducibility of NMR analysis of urine samples: impact of sample preparation, storage conditions and animal health status

Christina Schreier[1], Werner Kremer[1,2], Fritz Huber[1], Sindy Neumann[1], Philipp Pagel[1], Kai Lienemann[3], Sabine Pestel[4,*]

SUPPLEMENT

Supplement table 1: measured pH values of the stability test samples

		Natrosol	Dilution [Δ%]	Furosemide	Dilution [Δ%]	HCBD	Dilution [Δ%]
**Original Sample**	Before	6.40	-	6.63	-	6.79	-
	After	7.06		7.25		7.25	
**pH 3**	Before	2.90	5.56	2.94	2.99	2.90	5.45
	After	6.62		6.92		6.78	
**pH 4**	Before	3.83	3.90	3.99	1.89	4.04	3.35
	After	6.75		7.05		6.95	
**pH 5**	Before	4.76	2.35	5.05	0.98	5.00	1.89
	After	6.87		7.15		7.08	
**pH 6**	Before	5.97	0.64	6.04	0.41	6.02	0.91
	After	6.92		7.21		7.17	
**pH 6.5**	Before	6.47	0.39	6.41	0.15	6.49	0.38
	After	7.11		7.25		7.23	
**pH 7**	Before	6.96	1.72	6.95	0.15	6.96	0.27
	After	7.23		7.29		7.30	
**pH 7.5**	Before	7.32	2.68	7.46	0.38	7.49	0.91
	After	7.34		7.31		7.38	
**pH 8**	Before	8.18	4.26	7.90	0.61	8.02	1.37
	After	7.48		7.34		7.42	
**pH 9**	Before	8.84	5.11	9.15	1.54	9.02	2.62
	After	7.64		7.47		7.57	

Rats were treated with natrosol, furosemide or HCBD and urine was collected at 0 - 8 h for furosemide and at 8 - 24 h for natrosol and HCBD. Original samples were titrated with 1 N NaOH or 1 N HCl according to a previously defined algorithm to achieve the target pH. Achieved pH values of the samples were measured before and after addition of phosphate buffer used for sample preparation for ^{1}H NMR measurements. Additionally, dilution factors from the addition of 1 N NaOH or 1 N HCl are calculated in % deviation as compared to the original sample.

Supplement table 2: salt concentrations and measured osmolality of the stability test samples

	Concentration / Osmolality	Natrosol	Furosemide	HCBD	Dilution [Δ%]
**Original Sample**	Na^+ [mmol/L]	24	87	17	-
	Cl^- [mmol/L]	45	152	45	
	Osm [mosm/L]	562	403	477	
**2-fold**	Na^+ [mmol/L]	74	136	67	0.84
	Cl^- [mmol/L]	95	201	95	
	Osm [mosm/L]	634	469	522	
**3-fold**	Na^+ [mmol/L]	124	186	117	1.67
	Cl^- [mmol/L]	145	250	145	
	Osm [mosm/L]	704	517	620	
**4-fold**	Na^+ [mmol/L]	174	235	167	2.51
	Cl^- [mmol/L]	195	299	195	
	Osm [mosm/L]	764	589	771	
**6-fold**	Na^+ [mmol/L]	272	333	266	4.16
	Cl^- [mmol/L]	293	396	293	
	Osm [mosm/L]	916	713	911	
**8-fold**	Na^+ [mmol/L]	372	431	365	5.82
	Cl^- [mmol/L]	392	492	392	
	Osm [mosm/L]	1028	848	993	
**10-fold**	Na^+ [mmol/L]	471	529	465	7.48
	Cl^- [mmol/L]	491	590	491	
	Osm [mosm/L]	1178	980	n.a.	
**15-fold**	Na^+ [mmol/L]	719	775	713	11.64
	Cl^- [mmol/L]	7388	833	738	
	Osm [mosm/L]	1529	1307	1765	
**20-fold**	Na^+ [mmol/L]	971	1024	965	15.85
	Cl^- [mmol/L]	989	1079	989	
	Osm [mosm/L]	1900	1696	1803	

Rats were treated with natrosol, furosemide or HCBD and urine was collected at 0 - 8 h for furosemide and at 8 - 24 h for natrosol and HCBD. Na^+ and Cl^- concentrations were measured in the original samples, and were calculated for the modified samples, since high electrolyte concentrations can not be measured directly. Therefore, osmolality was measured in all samples as a surrogate for electrolyte (Na^+ and Cl^-) measurements. Dilution factors from addition of 35 % NaCl solution are calculated as relative deviation from the original sample in % (Δ%). Abbreviation: n.a. not available

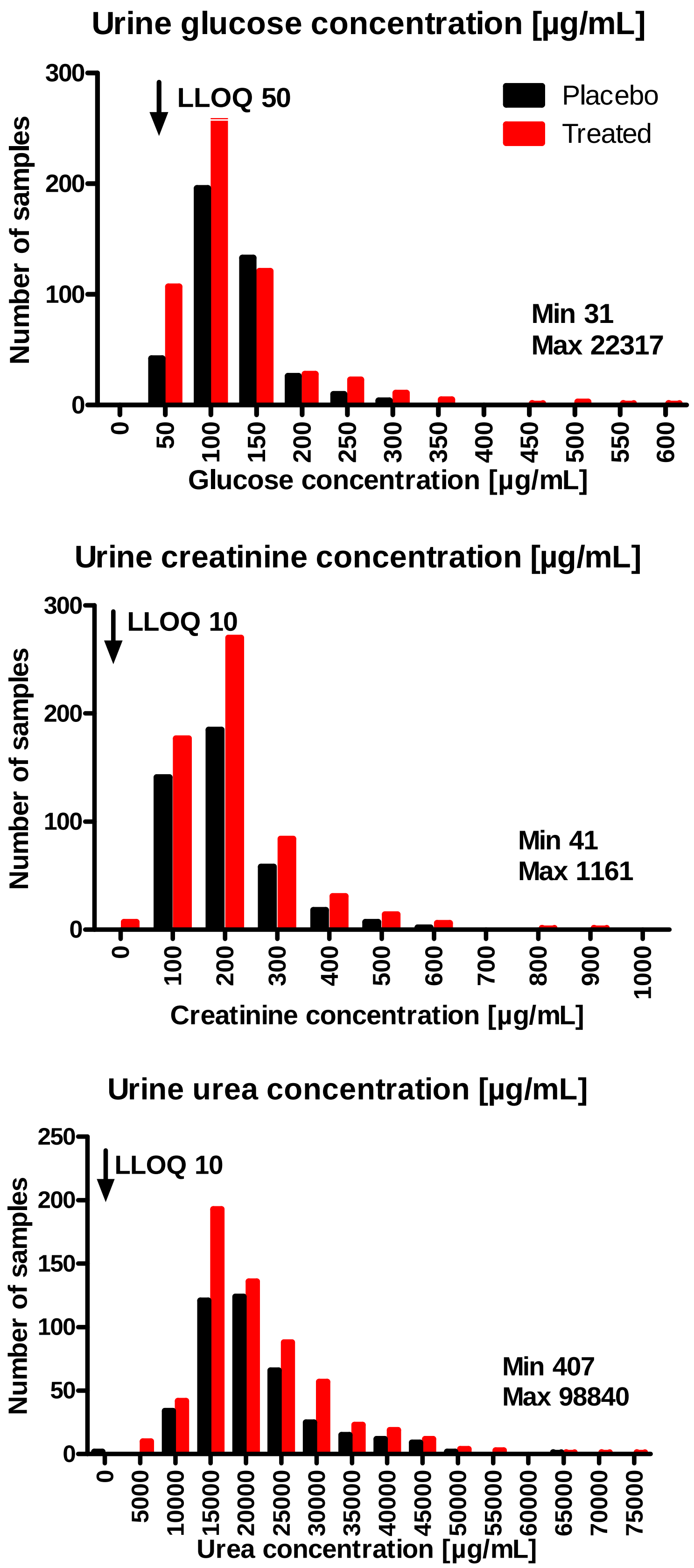
Urine glucose concentration [µg/mL]
LLOQ 50
Placebo
Treated
Min 31
Max 22317
Number of samples
Glucose concentration [µg/mL]
Urine creatinine concentration [µg/mL]
LLOQ 10
Min 41
Max 1161
Number of samples
Creatinine concentration [µg/mL]
Urine urea concentration [µg/mL]
LLOQ 10
Min 407
Max 98840
Number of samples
Urea concentration [µg/mL]

Supplement Figure 1: glucose, creatinine and urea concentrations in rat urine

Male and female rats were treated with natrosol, furosemide, formoterol, spironolactone, amiloride, acetaminophen, adriamycine or hydrochlorothiazide and urine was collected at 0 - 4 h, 4 - 8 h and 8 - 24 h post administration. Rats were fed before the experiment and fasted during the experiment, leading to a mixture of urine samples from fed and fasted animals. Glucose, creatinine and urea concentrations were measured, and histograms were plotted showing normal and pharmacological ranges of the parameters. Data include samples from 411 vehicle-treated animals and 592 - 593 reference compound-treated animals.

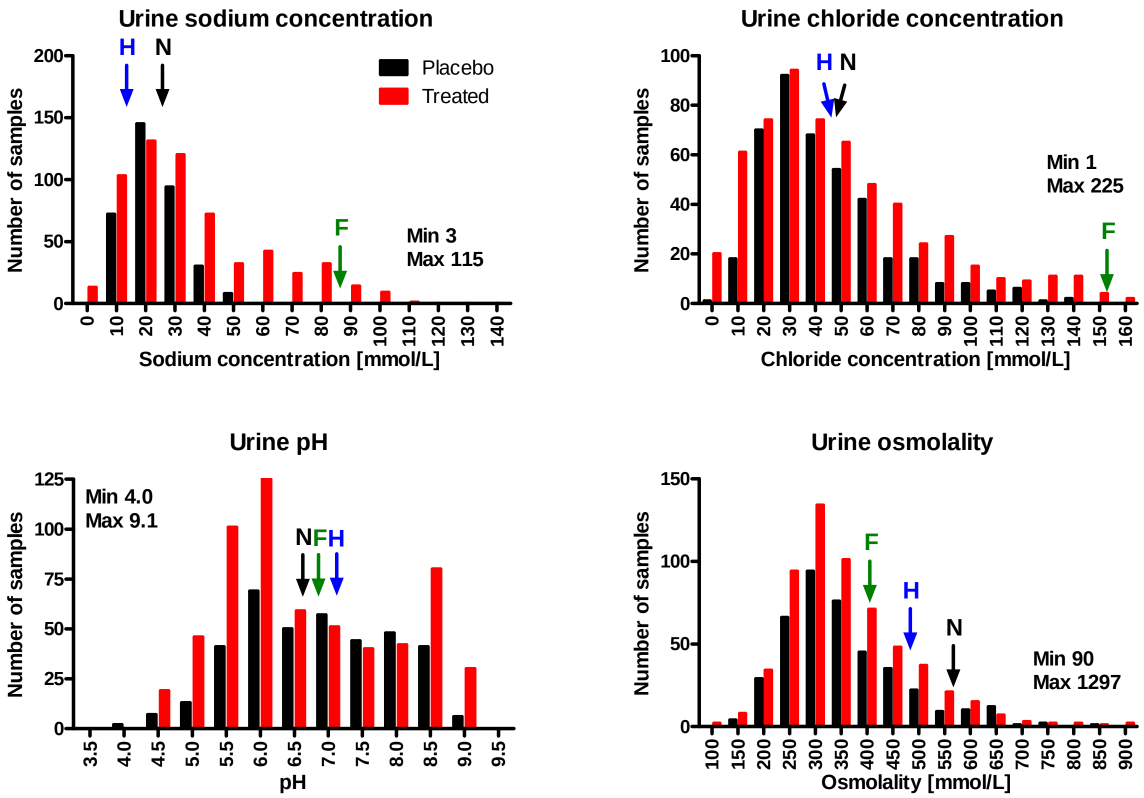

Supplement Figure 2: sodium and chloride concentrations, pH and osmolality in rat urine

Male and female rats were treated with natrosol, furosemide, formoterol, spironolactone, amiloride, acetaminophen, adriamycin or hydrochlorothiazide and urine was collected at 0 - 4 h, 4 - 8 h and 8 - 24 h post administration. Rats were fed before the experiment and fasted during the experiment, leading to a mixture of urine samples from fed and fasted animals. Na^+ and Cl^- concentrations, pH and osmolality were measured, and histograms were plotted showing normal and pharmacological ranges of the parameters. Data include samples from 349 - 411 vehicle-treated animals and 582 - 589 reference compound-treated animals. Additionally, values from the original samples of the stability test are plotted. Abbreviations: N natrosol, F furosemide, H HCBD.
